# Climate change and infectious diseases: breaking the poverty-disease-environment trap

**DOI:** 10.1186/s40249-026-01455-4

**Published:** 2026-05-10

**Authors:** Mehdi Borhani, Vahid Nourani, Hao Wen

**Affiliations:** 1 State Key Laboratory of Pathogenesis, Prevention and Treatment of High Incidence Diseases in Central Asia, Xinjiang Medical University, Urumqi, China; 2 Joint Key Laboratory for International Cooperation on Major Diseases in Central Asia, Xinjiang Medical University, Urumqi, China; 3 Faculty of Civil Engineering, University of Tabriz, Tabriz, East Azerbaijan Iran; 4 Altinbas University, Sht. Kemal Ali Omer St. No:22 YenisehirVia Mersin 10, Nicosia, Turkey; 5 Department of Hepatobiliary and Hydatid Surgery, The First Affiliated Hospital of Xinjiang Medical University, Urumqi, China

**Keywords:** Climate change, Infectious diseases, One Health, Poverty

## Abstract

Climate change and infectious diseases jointly form a self-reinforcing poverty disease environment trap that disproportionately harms impoverished populations in low- and middle-income countries. Climate change amplifies the transmission of vector -borne, water-borne, and zoonotic infectious diseases by expanding vector distribution, triggering water source contamination via extreme weather, and disrupting ecological balance. Poverty worsens vulnerability due to insufficient health infrastructure, limited resource access, and weak adaptive capacity, with sub-Saharan Africa and South Asia facing the highest risks. Climate related health harms are projected to push 44 million people into extreme poverty by 2030. This correspondence aims to clarify the linkages between climate change, infectious diseases, and poverty, and propose integrated solutions to break the vicious cycle. Effective interventions include building climate resilient health systems, addressing social determinants of health vulnerability, and adopting cross sectoral One Health collaboration. This work highlights climate change as a critical public health and equity issue, calling for integrated and equitable actions to protect vulnerable groups and sustain global health progress.

## Introduction

The intertwined crises of climate change and infectious diseases have emerged as defining challenges of the twenty-first century, with their most severe impacts disproportionately experienced by populations living in poverty. Human induced climate change is one of the greatest threats to human health, affecting food and water security, livelihoods, migration and displacement, mental health, and the transmission of infectious diseases [1, 2].

Extreme climate events, including droughts, floods, tropical cyclones, and heat waves, are rising in frequency and intensity, with growing impacts on human health [2]. Exploring this connection is not merely an academic exercise; it is a prerequisite for designing equitable and effective public health interventions. Climate change acts as a powerful amplifier of infectious disease transmission, while poverty exacerbates vulnerability to climate shocks and disease outbreaks, creating a self reinforcing cycle that threatens to reverse decades of global health progress. Addressing this complex interaction requires a paradigm shift from disease control to integrated, climate resilient health systems based on the principles of One Health and social justice.

## Pathways linking climate change to infectious diseases

Climate change is changing the epidemiology of infectious diseases through multiple and interconnected pathways, with vector-borne, water-borne, and zoonotic diseases most affected [1, 3, 4].

Rising global temperatures have expanded the geographic range and seasonal activity of vector species such as *Aedes* mosquitoes (which carry dengue and chikungunya) and *Anopheles* mosquitoes (which carry malaria), enabling these pathogens to expand into new geographic areas where they were not previously native, in temperate regions and at higher altitudes [5–7]. Extreme weather events including floods, droughts and heat waves further exacerbate disease risks; floods contaminate water sources and drive cholera and typhoid outbreaks, while droughts limit access to safe drinking water and aggravate malnutrition, a major risk factor for tuberculosis (TB) [8]. In addition, climate induced environmental degradation and human displacement disrupt ecological balances, increase human wildlife interactions, and facilitate the transmission of pathogens shared between humans and animals [9].

Numerous studies have documented that COVID-19, Ebola, AIDS, avian influenza and malaria, which are major emerging infectious diseases, are due to human interference with ecosystems, such as deforestation and urbanization, which have disrupted ecological balances, increased human wildlife interactions and exacerbated the spread of pathogens between species [1, 10, 11].

## Regional inequities and vulnerable populations

Human mortality from extreme climate events is 15 times higher in areas of high vulnerability than in areas of very low vulnerability [2]. The Global South and low income countries, especially indigenous peoples and local communities, continue to disproportionately experience climate change impacts. Historical and structural inequalities have amplified vulnerability to infectious diseases and climate disasters [12].

Even in large emerging economies like China, insufficient local community engagement leaves populations more exposed to climate exacerbated zoonotic and infectious disease risks [13]. These systemic weaknesses mirror global inequities; vulnerable groups lack consistent protection because health, animal, and environmental systems operate in silos rather than through integrated One Health frameworks [14].

Climate change adaptation strategies are urgently needed to address this growing and unequal health crisis [15]. Structural factors include extractive economic systems, underinvestment in local health systems, unequal resource distribution, and weak governance structures that persist in many Global South nations, reducing adaptive capacity and amplifying health vulnerability.

### Case examples: high risk regions

For instance, the Middle East and North Africa (MENA) region one of the world’s most climate vulnerable zones faces rising temperatures, water scarcity, and more frequent heatwaves and droughts that expand tick habitats and boost transmission of tick-borne zoonotic diseases such as Crimean Congo hemorrhagic fever (CCHF) and bovine theileriosis. Livestock herders, slaughterhouse workers, and rural communities in Iraq, Tunisia, and the UAE bear disproportionate risk, with CCHF mortality rates reaching 17–29% in affected populations, while limited surveillance and healthcare access worsen outcomes for marginalized groups [16–18]. These disparities reflect structural inequities that amplify climate driven infectious disease risk in the Global South [3].

In Nigeria, one of the world’s most climate vulnerable nations, rising temperatures, erratic rainfall, droughts, and floods have severely disrupted rain fed agriculture which supports over 70% of the population [19]. Smallholder farmers, rural households, and low income communities face falling crop yields, rising food prices, increased pest and disease pressure, and resource conflicts that deepen inequality and health risks. Lake Chad has shrunk from 40,000 km^2^ to just 1300 km^2^, destroying livelihoods for fishing and pastoral communities and worsening food insecurity and displacement [20].

## The poverty disease environment trap: a vicious cycle

Poverty exacerbates the health impacts of climate related infectious diseases through multiple structural vulnerabilities. Beyond limited access to early diagnosis and treatment, low- and middle-income countries (LMICs) often lack robust health infrastructure, adequate sanitation, safe housing conditions, and planned land use, all of which directly elevate exposure to water-borne, vector-borne, and zoonotic pathogens [21]. Poor households also face persistent barriers to education, stable livelihoods, and social protection, with low educational attainment reducing health literacy, limiting adoption of preventive behaviors, and weakening the ability to respond to disease outbreaks. These economic and political determinants of health further reduce adaptive capacity and increase disease risk. Climate shocks damage clinics, disrupt vaccine supply chains, and interrupt care for chronic infectious diseases such as TB.

The intersection of the climate crisis, extreme poverty, and public health vulnerabilities is emerging as a critical global challenge, with profound implications for achieving the Sustainable Development Goals, particularly those related to poverty eradication and good health and well being. The climate crisis is projected to push 44 million people into extreme poverty by 2030, a figure that is clearly driven by adverse health impacts [22]. This finding underscores the need to consider climate change not only as an environmental issue, but also as a major public health and equity concern, given its disproportionate impacts on marginalized populations. Notably, the report identifies women, children, the elderly, ethnic minorities, people with underlying health conditions, and those already living in poverty as the most vulnerable groups to climate related health risks. This vulnerability is more geographically concentrated, with sub Saharan Africa and South Asia projected to disproportionately experience extreme poverty from climate change, with an additional 132 million people in these regions at risk of poverty by 2030 [22].

This regional concentration reflects the confluence of existing socioeconomic inequalities, limited adaptive capacity, and increased exposure to climate hazards factors that reinforce the poverty health feedback loop. This creates a vicious poverty disease environment trap; the spread of climate induced diseases reduces agricultural production and labor force capacity, and forces households to overexploit natural resources (e.g., deforestation for fuel or agriculture), further degrading ecosystems and increasing the risks of disease transmission. Compounding these challenges, marginalized groups including displaced people, women and children face increased exposure to infectious diseases due to limited access to health, healthcare and social protection.

## Integrated strategies to break the cycle

**Fig. 1:**
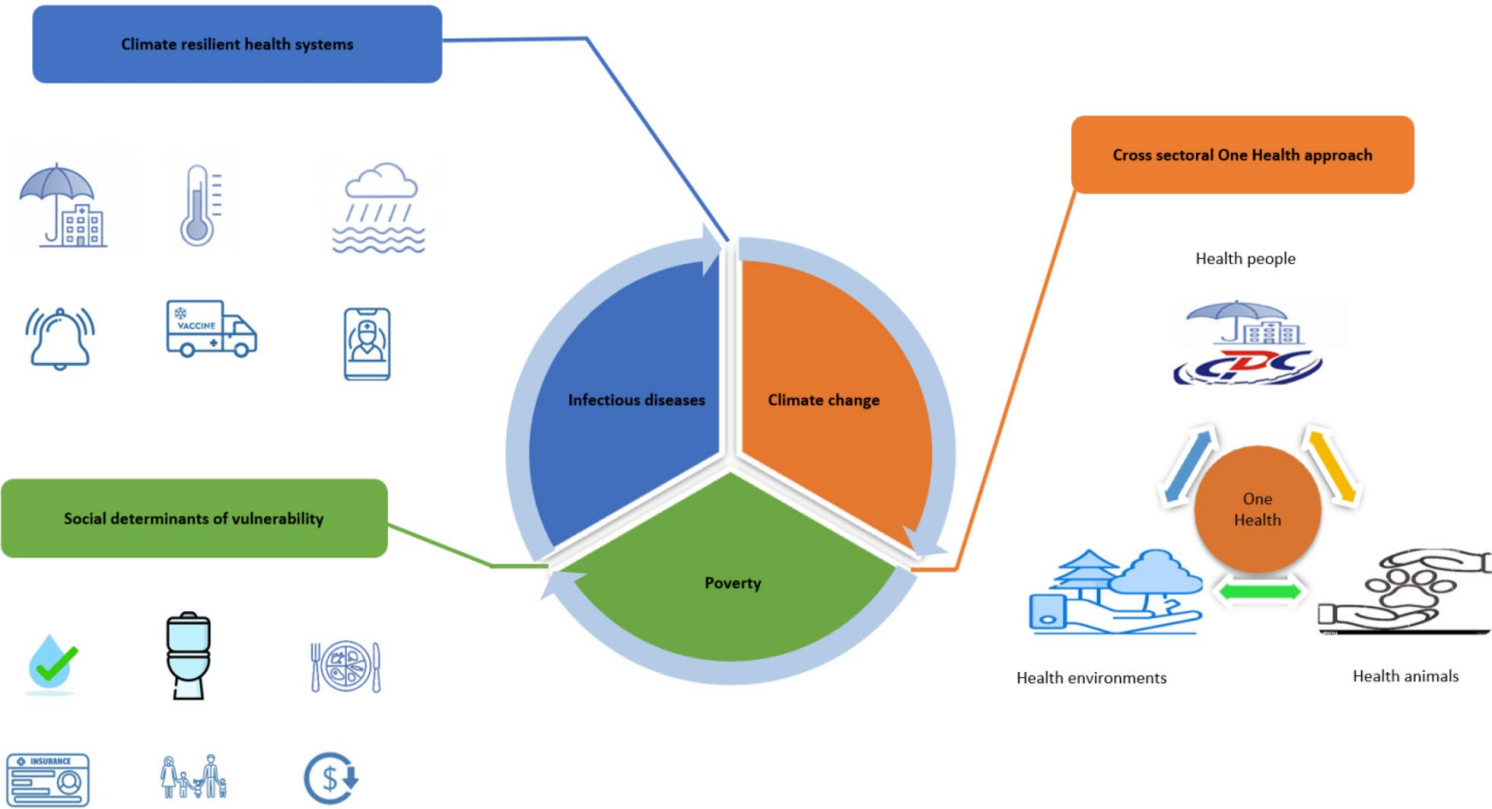
Schematic figure of the main integrated strategies for breaking the environmental trap of the disease of poverty

The three core integrated strategies to break the poverty disease environment trap are summarized below and illustrated in Table. 1 and Fig. 1.

**Table. 1:** Three core strategies to break the poverty disease environment trap amid climate change and infectious disease risks

Strategy 1 Strengthen LMICs health systems to withstand climate shocks	1. Deploy climate resilient modular health infrastructure. 2. Establish decentralized climate health surveillance hubs. 3. Develop climate shock response protocols for health facilities.
Strategy 2 Address social determinants of vulnerability to climate-infectious disease risks	1. Scale targeted financial risk protection mechanisms 2. Implement community led WASH infrastructure projects. 3. Launch climate smart nutrition programs for high risk groups. 4. Advocate for equitable land tenure and livelihood support.
Strategy 3 Implement a One Health approach to address ecological drivers of diseases transmission	1. Establish cross sectoral One Health surveillance task forces. 2. Promote sustainable agricultural practices via incentive schemes. 3. Scale integrated vector management (IVM) programs. 4. Facilitate cross border One Health collaboration for migratory species and populations.

These integrated interventions aim to build equity and resilience, protecting vulnerable populations and safeguarding global health gains. *LMICs* Low and Middle Income Countries, *WASH* Water, Sanitation, Hygiene.

Climate resilient health systems are defined as health systems designed to prevent, prepare for, absorb, and recover from climate shocks while maintaining equitable service delivery, disease surveillance, and emergency response. Addressing the nexus between climate, infectious diseases and poverty requires integrated, cross sectoral strategies that prioritize equity and resilience.

### Strengthen climate resilient health systems in LMICs

Health systems in LMICs need to be strengthened to withstand climate shocks, by investing in climate resilient infrastructure, decentralized health service delivery and robust surveillance systems that integrate climate data for early warning of disease outbreaks. Strengthening health systems in the Global South specifically targets resilience against climate shocks including floods, heatwaves, and droughts. Strengthening these systems in LMICs means retrofitting clinics, stabilizing supply chains, and training frontline workers to manage climate shocks, ensuring uninterrupted care for vulnerable groups.

Climate resilient health infrastructure includes elevated outpatient clinics, flood proof storage for medicines and vaccines, and on site backup power systems. Decentralized delivery uses mobile health units and community health workers to reach remote areas. These investments add value by expanding geographic access, maintaining services during climate disasters, and reducing reliance on overstretched central hospitals.

Robust surveillance systems integrated with real time climate data enable data driven early warning for vector-borne, water-borne, and zoonotic disease outbreaks. Temperature, rainfall, and humidity data predict vector expansion and water contamination risks. Implementation uses digital surveillance platforms and cross agency data sharing. The key added value is shifting from reactive response to proactive prevention, reducing outbreak scale and saving lives in high risk regions.

### Address social determinants of vulnerability

Interventions must address the social determinants of vulnerability, including expanding universal health coverage, reducing out of pocket health care costs, and improving access to clean water, sanitation, and nutrition. Addressing social determinants targets root vulnerabilities; universal health coverage reduces financial barriers to care; clean water and sanitation eliminate water-borne disease transmission; improved nutrition boosts immune resistance to infections. Implementation includes policy reforms, subsidized essential services, and community led Water, Sanitation, Hygiene (WASH) programs. This approach adds value by breaking the structural links between poverty, poor living conditions, and climate sensitive infectious diseases.

### Adopt a cross sectoral One Health approach

A cross sectoral One Health approach that unites health, environment, agriculture, and migration sectors is essential to target the ecological origins of disease transmission. This framework supports joint surveillance at the human animal environment interface, promotes sustainable agriculture to reduce ecosystem disruption, and enables cross border coordination for mobile populations. This strategy adds unique value by addressing the ecological origins of emerging zoonotic diseases, which single sector health interventions cannot achieve. This includes strengthening surveillance of zoonotic pathogens at the human animal environment interface, promoting sustainable agricultural practices that reduce ecosystem degradation, and managing vector habitats through integrated pest management rather than over reliance on chemical pesticides.

International cooperation is critical to support these efforts, as high income countries meet their commitments to climate finance and technology transfer to lLMICs. Furthermore, empowering researchers and local communities in disease endemic areas to design tailored solutions is critical. Targeted research and innovation in the Global South includes developing low cost climate resilient health technologies, regional disease risk mapping, and context‑adapted intervention designs. Implementation involves strengthening local research capacity and knowledge exchange. The added value is generating locally relevant evidence to avoid one size fits all interventions, improving scalability and long‑term success in resource limited settings.

## Governance, financing, and scalable low cost models

Successful implementation relies on clear governance structures and sustainable financing mechanisms to translate policy recommendations into practice. High income countries must deliver on their climate finance commitments and facilitate equitable technology transfer to LMICs, while local governments and multilateral institutions including the World Health Organization (WHO) and World Bank take lead responsibility for on the ground delivery in the most vulnerable regions. To build sustainable capacity, scalable grassroots models include community led disease surveillance, targeted vector control, and local safe water management committees. Empowering local researchers and community stakeholders further ensures interventions are culturally appropriate, locally owned, and sustainable over the long term.

Successful low cost models exist in LMICs; decentralized One Health hubs in East Africa improve zoonotic disease detection [23].

Practical, low cost interventions include community health worker led surveillance, solar powered rural clinics, rainwater harvesting for safe water, school based nutrition programs, and cross sectoral One Health task forces sharing data across human, animal, and environmental sectors.

## Evidence from successful interventions

Integrated One Health strategies have been successfully implemented in LMICs, providing actionable models for climate resilient health systems. For example, Brazil has established a national climate health surveillance system that integrates meteorological data to provide early warning for dengue and malaria outbreaks, demonstrating how cross sectoral collaboration can strengthen disease prevention and control in resource limited settings [24]. Similar community led and decentralized One Health models across South Asia and sub Saharan Africa further validate the real world impact of these approaches, highlighting the feasibility of scaling equitable, context appropriate public health policies aligned with global health goals.

Community led WASH programs in Bangladesh reduced cholera and diarrheal disease risk in flood prone and high transmission slum settings. The hospital based and community WASH intervention cut cholera infections among household contacts by 47% and reduced overall diarrhea prevalence in at risk groups, demonstrating the impact of targeted WASH delivery in vulnerable urban and flood affected communities [25].

Lapata et al. (2024) provided strong evidence for the success of One Health in controlling soil transmitted helminthiases (STHs), a major neglected tropical disease. Their work showed that integrating mass drug administration (MDA) with One Health components including improved water and sanitation, hygiene promotion, health education, and environmental management produced sustained reductions in STH prevalence. Specifically, water interventions reduced hookworm by 31%, sanitation reduced overall STH by 29%, and health education lowered infection rates by 58% in school aged children. This integrated model surpasses standalone deworming programs by addressing the environmental and behavioral drivers of transmission, validating One Health as a critical strategy for sustainable STH elimination [26].

## Conclusions

Consequently, climate change is not only an environmental crisis, but a major threat to global health equity, and its impacts on infectious diseases disproportionately affect the world’s poor. Breaking this cycle requires shifting from reactive disease control to building climate resilient health systems, reducing structural inequalities, and strengthening cross sectoral collaboration [27]. As the global community grapples with the climate crisis and the continuing burden of infectious diseases, the global scientific and public health communities play a critical role in advancing research that links environmental science, public health, and development.

## Data Availability

All data and materials referenced in this manuscript are derived from publicly available sources, including peer reviewed literature, international organization reports, and academic databases. References for all cited sources are provided in the References section. No unpublished data or proprietary materials were used.

## Abbreviations

LMICs: Low and middle income Countries
TB: Tuberculosis
WASH: Water, Sanitation, Hygiene
STHs: Soil transmitted helminthiases
CCHF: Crimean Congo hemorrhagic fever
MENA: Middle East and North Africa
WHO: World Health Organization
MAD: Mass drug administration
